# Metabolomic Differential Compounds Reflecting the Clinical Efficacy of Polyethylene Glycol Recombinant Human Growth Hormone in the Treatment of Childhood Growth Hormone Deficiency

**DOI:** 10.3389/fphar.2022.864058

**Published:** 2022-04-27

**Authors:** Ji Li, Weiwei Pan, Jianqin Qian, Yan Ni, Junfen Fu, Shaoqing Ni

**Affiliations:** ^1^ National Clinical Trial Institute, The Children’s Hospital, Zhejiang University School of Medicine, National Clinical Research Center for Child Health, Hangzhou, China; ^2^ Department of Pharmacy, Children’s Hospital, Zhejiang University School of Medicine, Hangzhou, China; ^3^ The Children’s Hospital, Zhejiang University School of Medicine, National Clinical Research Center for Child Health, Hangzhou, China; ^4^ Department of Endocrinology, Children’s Hospital, Zhejiang University School of Medicine, Hangzhou, China; ^5^ Research Center for Clinical Pharmacy, Zhejiang University, Hangzhou, China

**Keywords:** growth hormone deficiency, PEG-rhGH, metabolomics, clinical efficacy, biomarkers

## Abstract

Understanding metabolite profiles may aid in providing a reference for individualized treatment using PEG-rhGH. Therefore, this study aimed to evaluate the clinical efficacy of PEG-rhGH in treating GHD patients by using a metabolomic approach. Fifty-seven pediatric participants treated with PEG-rhGH were enrolled (28 GHD patients with high clinical efficacy and 29 GHD patients with lower clinical efficacy). Serum samples from all patients were first collected at baseline for biochemical detection; then metabolite levels were measured using gas chromatography time-of-flight mass spectrometry. The candidates included heptadecanoic acid, stearic acid, 2-hydroxybutyric acid, myristic acid, palmitoleic acid, D-galactose, dodecanoic acid, and oleic acid. The related metabolic pathways involved fatty acid metabolism and energy metabolism. This study suggested that growth gains of PEG-rhGH treatment might be differentiated by altered serum levels of fatty acid. Collectively, the metabolomic study provides unique insights into the use of PEG-rhGH as a therapeutic strategy for individualized treatment.

## Introduction

The prevalence of growth hormone deficiency (GHD) in children is on the rise worldwide. The focus on children’s health is more urgent owing to their estimated greater vulnerability (Jee et al., 2017; Murray et al., 2018; Halas and Grimberg, 2020). GHD is a developmental disorder caused by either partial or complete deficiency in the synthesis and secretion of growth hormone in the anterior pituitary lobe or by receptor defects and structural abnormalities. Growth failure, the primary apparent feature of GHD, may influence the life quality and psychosocial development of affected children (Chaplin et al., 2015; Chinoy and Murray, 2016; Leonibus et al., 2016). As recorded in recent pharmaceutical studies, recombinant human growth hormone (rhGH) showed a significant therapeutic effect against growth hormone deficiency (Rogol et al., 2013). To achieve this effect, rhGH is required to be administered as a daily injection (López-Siguero et al., 2011; Wit et al., 2013). Frequent injections cause distress to the children and therefore reduce compliance. PEG-rhGH preparation is a covalent conjugate of rhGH and branched polyethylene glycol (PEG), which can potentially increase the molecular weight of rhGH, reduce drug toxicity and prolong the half-life of elimination *in vivo* (Cutfield et al., 2011; Rogol et al., 2013; Lundberg et al., 2018; Wang et al., 2021). The treatment of polyethylene glycol-modified long-acting rhGH (PEG-rhGH) requires only weekly injections, which can reduce the frequency of injections and can enhance children’s compliance with rhGH treatment.

However, to the best of our knowledge, relatively little is known about the metabolic changes that are associated with differences in clinical efficacy of PEG-rhGH replacement therapy (Rasmussen et al., 2010; Schepper et al., 2011; Hou et al., 2015). Therefore, it is of interest to find predictive biological metabolites for monitoring individual responses to PEG-rhGH therapy. Metabolomics has stood out for providing rapid, sensitive, and less invasive analyses that identify and quantify endogenous metabolites present in different biological matrices (McBride et al., 2019). Studies on the disease diagnosis and treatment of rhGH by metabolomics have been reported in recent years (Rahman et al., 2013; Höybye et al., 2014; Xu et al., 2019). Research in 10 adults with GHD demonstrated that the level of serum metabolite was altered in GHD patients and that some specific fatty acid compounds and amino acids had the potential ability to be biomarkers for GHD. (Höybye et al., 2014). Metabolomics may be a powerful method for studying the clinical efficacy of PEG-rhGH intervention in improving GHD. This kind of improvement will be particularly useful for the pediatric population.

The primary objectives of the present study were to investigate the associations between clinical efficacy and metabolites in GHD children treated with PEG-rhGH and to find biomarkers and related metabolic pathways by metabolomic techniques, which are expected to provide a reference for the individualized treatment of PEG-rhGH.

## Material and Methods

### Patients and Samples

The present study was conducted on the basis of the multi-center drug clinical study project “Phase IV clinical trial of polyethylene glycol-recombinant human growth hormone injection in treating children growth hormone deficiency” led by the Children’s Hospital, Zhejiang University School of Medicine, which was registered at www.clinicaltrials.gov (NCT02314676). Sixty subjects with different clinical efficacies of PEG-rhGH treatment were randomly screened from the established PEG-rhGH phase IV clinical trial database, and three of these subjects were excluded due to incomplete data. The study was approved by the Ethics Committee of the Children’s Hospital, Zhejiang University School of Medicine and was conducted in accordance with the Declaration of Helsinki. All patients provided written informed consent (2018-IRB-033). The actual height of the patients after treatment was compared with the mean height of the population and its standard deviation (SD) of height for a chronological age, which was considered the primary outcome measure (△Ht SDS). The mean and SD value for height were calculated using reference values with The National Growth Survey of Children under 7 years in the Nine Cities of China in 2005. Patients were divided into two groups using the median △Ht SDS as a cutoff: △Ht SDS >0.44 (high clinical efficacy (HE) group, n = 28), and △Ht SDS ≤0.44 (low clinical efficacy (LE) group, n = 29). Patient inclusion criteria were as follows: diagnosed as GHD based on medical history, clinical symptoms, and signs; GH activation test and imaging criteria before treatment (height below the third percentile of the normal growth curve for children of the same age and sex at age of 2–18, rate of height increase ≤5.0 cm year^−1^, two-drug GH stimulation tests with different mechanisms of action confirmed that the plasma GH peak was less than 10.0 ng ml^−1^, for girls ≤9 years old and boys ≤10 years old, the bone age is more than 1 year behind the actual age, that is, the actual age minus bone age ≥1 year); pre-pubertal stage (Tanner I stage), age ≥3 years old, gender is not limited; did not receive growth hormone therapy within 6 months; and the subject is willing and able to complete the scheduled interview, treatment plan, laboratory examination and other procedures, and to sign a written informed consent. Exclusion criteria were as follows: abnormal liver and kidney function (ALT > two times the upper limit of normal (ULN), Cr > ULN); hepatitis B virus detection of HBc, HBsAg, and HBeAg were all positive; subjects with allergy constitution or allergy to the drug; subjects with severe CVD, lung diseases, hematological diseases, malignant cancer, systemic infections or a compromised immune system; subjects with underlying cancer (family history of cancer); subjects with diabetes; abnormal growth and development, such as Turner’s Syndrome, constitute delay of puberty; Laron Syndrome; growth hormone receptor deficiency; girls with short stature with chromosomal abnormalities; subjects participated in drug clinical trials within 3 months; subjects with positive anti-hGH antibodies; and other conditions that the investigator considers unsuitable for inclusion in this clinical trial.

### Sample Preparation

The metabolomic samples were analyzed with residual serum samples of IGF-1 and IGF-BP3 from 57 cases for freeze-thaw frequency less than 3 times. Samples were thawed at 4°C and were mixed adequately before analysis with chemical derivatization. A 50 µl aliquot of serum sample was spiked with 10 µl internal standard solutions (chlorophenyl alanine) and was vortexed for 10 s. The mixed solution was deproteinized using 200 µl of the extraction solvent (methanol: chloroform = 3:1 [v/v]). After 30 s of vertex, the samples were centrifuged at 13,500 rpm for 20 min at 4°C. A 200 µl supernatant was transferred to an autosampler vial, all samples in autosampler vials were evaporated by CentriVap vacuum concentrator for 5 min, and then they were further lyophilized with a cryogenic freeze-dryer (Labconco, Kansas City, Mo, United States). After the samples were vacuum-dried at room temperature under nitrogen, 50 µl of methoxyamine (20 mg ml^−1^ in pyridine) was added to each vial and kept at 30°C for 120 min, followed by 50 µl of MSTFA at 37.5°C for 60 min. This silylated derivation was performed using a Gerstel multipurpose sample MPS2 with dual heads (Gerstel GmbH and Co., Mulheim, Germany), and the derived samples were automatically injected into GC/TOFMS for metabolomics analysis.

### GC-TOFMS Analysis

Serum metabolite profile was acquired by time-of-flight mass spectrometry system (Pegasus HT, Leco Corp., St. Joseph, MO, United States) coupled with gas chromatography (Agilent 7890B, Santa Clara, CA, United States). The chromatographic separation of the serum sample was performed on an Rxi-5MS capillary column (30 m × 250 µm I.D., 0.25 µm). The column flow rate was set at 1.0 ml min^−1^, and helium was used as the carrier gas. The temperature programs were set up as follows: 80°C hold on for 2 min, then raised to 300°C at a rate of 12°C·min^−1^ for 4.5 min, and finally to 320°C at a rate of 40°Cmin^−1^ for 1 min. The temperatures for front injection, transfer line, and ion source were 270, 270, and 220°C, respectively. Electron impact ionization of 70 eV in the scan range (50–550 Da) was applied, and the acquisition rate was 20 spectra s^−1^. The instrument was optimized and maintained every 24 h to monitor its stability.

### GC-TOFMS Data Processing and Statistical Analysis

Raw data from GC/TOFMS analysis were processed using ChromaTOF software (v4.51.6.0, Leco., CA, United States) to achieve the following elements of preprocessing: baseline correction and smoothing, deconvolution, extraction, and alignment of original chromatographic peak signals, retention index correction, and metabolite identification using standard materials. The self-developed platform iMAP (v1.0, Metabo-Profile, Shanghai, China) was used for statistical analyses, including data preprocessing (normalization and standardization), statistical analysis (multidimensional statistical analysis and one-dimensional statistical analysis), metabolic network analysis, and reporting. Following preprocessing, the data were presented as mean ± standard deviation (SD). The *p*-value of 0.05 was considered to indicate statistical significance. The SIMCA 14.1 software (MKS Data Analytics Solutions, Umea, Sweden) was applied for principal component analysis (PCA) and orthogonal partial least squares discriminant analysis (OPLS-DA). Permutation tests were performed with 200 iterations to validate the model. The features were filtered initially by variable importance in the projection (VIP) value (VIP > 1) and fold change value (FC > 1) in the comparison of HE and LE patient groups. The Mann-Whitney *U* test was applied to assess the significant difference of these features between the groups. Differential serum metabolites were selected by considering VIP (>1.0), FC (>1.0) and *p*-value (<0.05). The receiver-operating characteristic (ROC) analysis was used to evaluate the specificity and sensitivity of potential biomarkers according to the area under the curve (AUC). Commercial databases, Kyoto Encyclopedia of Genes and Genomes (KEGG, http://www.genome.jp/kegg/) and Human Metabolome Database (http://www.hmdb.ca/) were utilized to search for the relative metabolites and metabolic pathways. A heatmap was used to show the association between clinical indicators and differential metabolites.

## Results

### Demographics and Serum Biochemical Analysis

The clinical characteristics of GHD patients at baseline were summarized in Table 1. Of the 60 GHD children that underwent baseline testing, 3 withdrew owing to incomplete data; therefore, data were reported from the 57 participants who completed the intervention. As anticipated, the improvement in bheight, height, weight, bone age, and age was lower in the HE group than in the LE group. Compared with the LE group, the level of total protein (TP, Table 1) in GHD patients of high clinical efficacy was significantly decreased. In addition, it was found that the differences in creatinine (CR, Table 1) and IGF-1 in the HE group were statistically significant compared to the LE group. The concentrations of other biochemical indexes did not differ significantly among treatments.

**TABLE 1:** Baseline clinical characteristics.

Item	LE group	HE group	^a^FC	^b^ *p*
^d^BHEIGHT (cm)	119.27 (13.79)	108.19 (10.12)	0.877	1.10 × 10^−3^
HEIGHT (cm)	121.8 (13.19)	110.98 (10.56)	0.885	1.70 × 10^−3^
WEIGHT (kg)	25.8 (7.38)	20.18 (5.96)	0.738	2.90 × 10^−3^
BONE AGE	6.81 (2.53)	4.79 (2.15)	0.571	3.30 × 10^−3^
AGE (year)	11.93 (2.79)	10 (2.29)	0.833	6.70 × 10^−3^
BMI	16.99 (2.38)	16.06 (2.24)	0.920	0.067
^c^SEX_STD	1.19 (0.40)	1.3 (0.47)	1.000	0.486
Liver function				
PHOS (mmol/L)	1.57 (0.19)	1.56 (0.16)	1.039	0.947
ALP (IU/L)	219.93 (71.59)	226.76 (58.69)	1.138	0.344
TBIL (μmol/L)	8.41 (5.34)	7.92 (2.95)	1.054	0.966
ALT (IU/L)	15.14 (6.02)	17.02 (7.76)	1.100	0.321
AST (IU/L)	29 (8.42)	31.17 (7.30)	1.089	0.174
ALB (g/L)	44.06 (2.96)	43.56 (2.63)	1.023	0.566
TP (g/L)	70.14 (3.95)	66.95 (4.82)	0.973	1.50 × 10^−2^
Renal function				
SG	1.02 (0.01)	1.02 (0.00)	1.001	0.611
BUN (mmol/L)	4.58 (0.97)	4.86 (1.25)	0.980	0.457
PH	6.4 (0.86)	6.25 (0.74)	0.923	0.442
CR (μmol/L)	47.93 (7.81)	41.24 (12.94)	0.856	1.80 × 10^−2^
UWBC	1.05 (1.09)	1.38 (1.85)	1.000	0.890
URBC	2.1 (2.31)	2.24 (3.46)	0.763	0.489
Blood lipid				
TG (mmol/L)	0.91 (0.62)	0.84 (0.34)	1.013	0.954
TC (mmol/L)	4.09 (0.68)	4.29 (0.99)	1.040	0.915
HDL (mmol/L)	1.52 (0.28)	1.45 (0.30)	0.980	0.561
LDL (mmol/L)	2.25 (0.54)	2.48 (0.77)	1.039	0.448
Blood glucose and hormone				
HBA1C (%)	5.26 (0.34)	5.31 (0.40)	1.000	0.574
GLU (mmol/L)	5.04 (1.06)	4.81 (0.40)	0.966	0.476
CORT (nmol/L)	97.68 (139.49)	130.75 (223.92)	1.196	0.634
ACTH (pmol/L)	31.63 (26.69)	25.25 (18.18)	1.043	0.595
INS (mIU/L)	14 (22.22)	9.27 (8.01)	0.805	0.574
Thyroid				
TSH (mIU/L)	2.91 (1.20)	2.81 (1.63)	0.936	0.560
T3 (nmol/L)	1.82 (0.49)	2.03 (0.50)	1.319	0.057
T4 (nmol/L)	70.07 (51.35)	82.65 (51.77)	1.135	0.242
Blood routine				
RBC ( × 10^12^/L)	4.7 (0.40)	4.62 (0.41)	0.951	0.212
WBC ( × 10^9^/L)	7.27 (2.16)	6.89 (2.28)	0.949	0.421
HCT	27.06 (20.39)	32.8 (13.12)	1.012	0.844
HB (g/L)	128.54 (11.64)	124.44 (9.16)	0.962	0.076
PLT ( × 10^9^/L)	283.08 (69.01)	293.3 (63.42)	1.007	0.588
Others				
IGF-1 (ng/ml)	174.63 (87.20)	111.4 (69.84)	0.595	4.80 × 10^−3^
IGFBP-3 (ng/ml)	3.54 (0.95)	2.96 (1.09)	0.880	0.076
CAL (mmol/L)	2.35 (0.19)	2.38 (0.13)	1.004	0.848

a FC, FC (fold change)-value. FC value is the multiple difference of the metabolite concentration between samples. A value less than 1 means that the metabolite content in HE group is lower than that in LE group, and a value greater than 1 means that the metabolite content in HE group is higher than that in LE group.

b p, p-value, which is obtained from Mann-Whitney U test, p < 0.05 means the difference is statistically significant.

c SEX_STD, 1 represents male and 2 represents female for gender comparison.

d BHEIGHT, height 1 year prior to the treatment.

### Metabolomic Profiling

**FIGURE 1:**
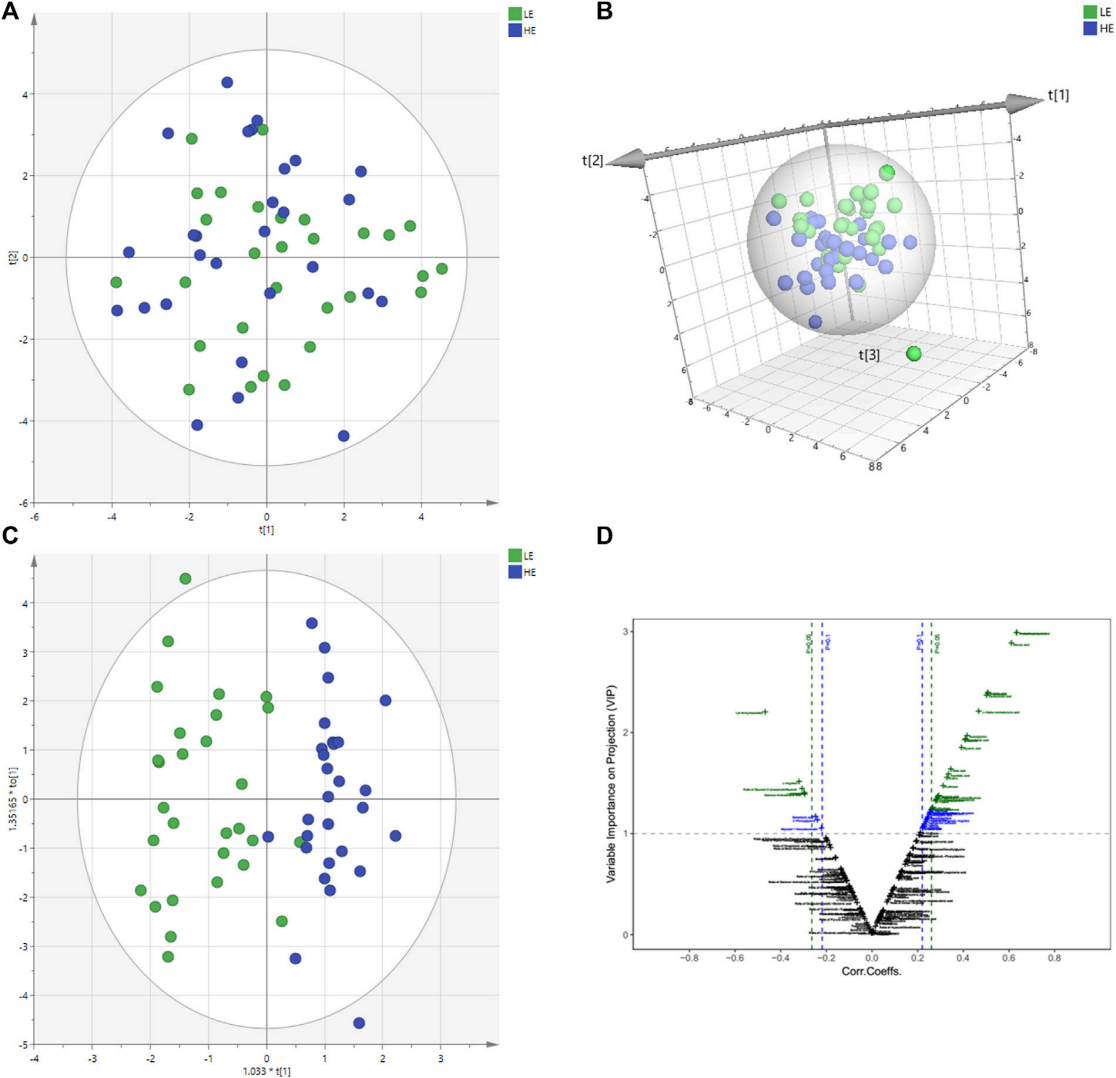
Metabolomics multivariate statistical analysis (MVA). PCA and PCA 3D score plots for pairwise comparisons between HE and LE from serum samples **(A,B)**. OPLS-DA score plots of HE group and LE group. LE (in green) represents low efficacy group and HE (in blue) represents high efficacy group. The clustering of HE group and LE group was obvious **(C)**. V-plot of differential metabolites **(D)**. On the left is the downregulated metabolites in the HE group compared with the LE group, and the correlation coefficient on the X-axis is negative. On the right is the up-regulated metabolites between the HE and the LE group, and the correlation coefficient on the X-axis is positive. HE, high clinical efficacy group; LE, low clinical efficacy group.

**FIGURE 2:**
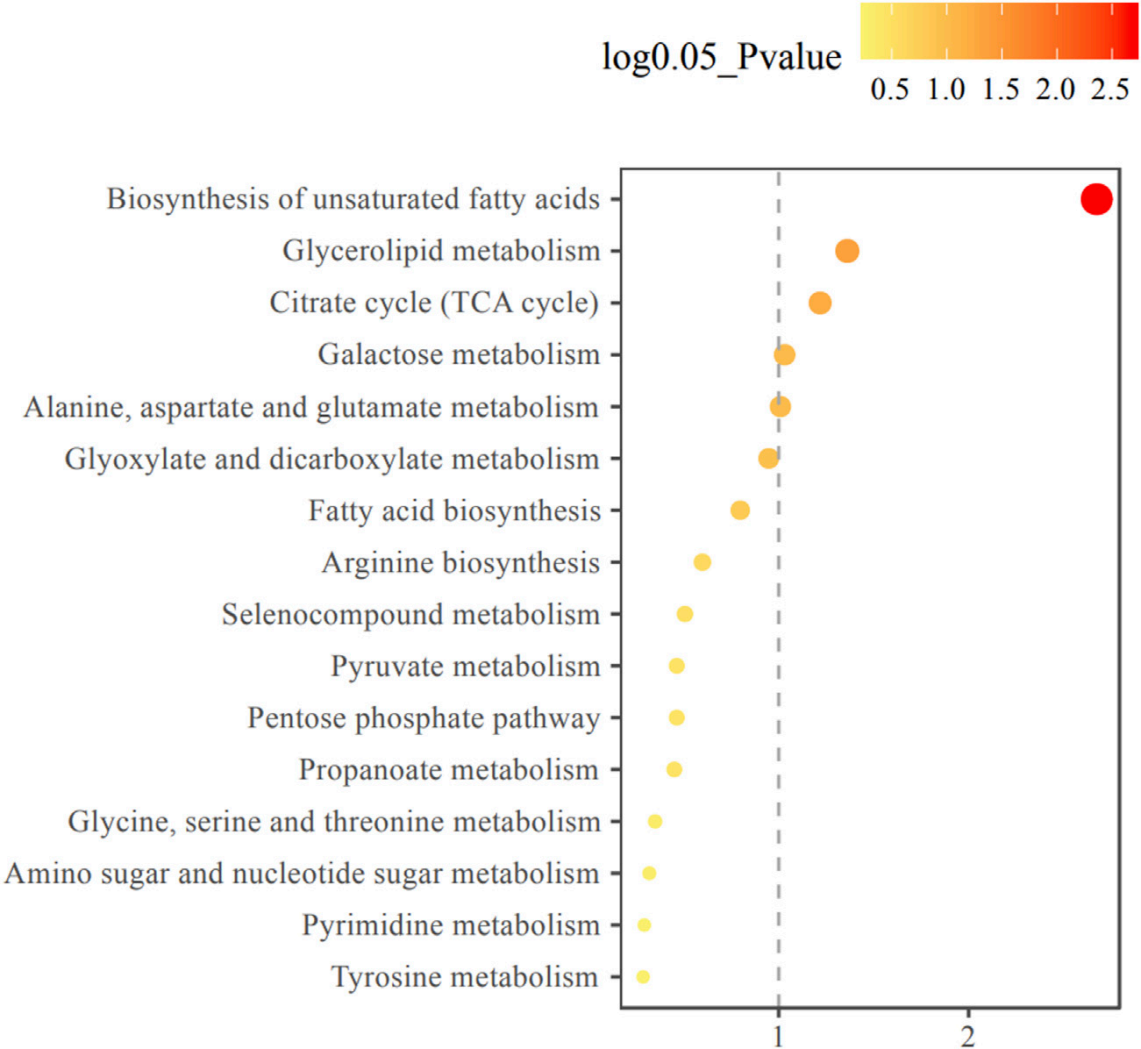
Metabolic pathway enrichment analysis diagram. Among the metabolic pathways associated with efficacy, there were statistically significant differences in glycerol lipid metabolism, TCA cycle, galactose metabolism, alanine, aspartate and glutamate metabolism pathways. The horizontal axis represents the enrichment factor and the vertical axis represents the pathway name. The color ranges from yellow to red, indicating that the adjusted *p*-value grows from small to large, and the enrichment degree becomes more and more significant. The size of the dot represents the number of metabolites enriched in this pathway.

The changes of metabolite in serum under different conditions could not be explored simply by visual inspection; thus, multivariate statistical analysis was applied to explore the differences in serum metabolites between groups. PCA, an unsupervised method, was used to obtain general clustering, trends, or outliers among the observations acquired in metabolic changes. The outline of the differences in serum metabolome was provided by an OPLS-DA model, which was used to identify the discriminant metabolic profiles and to determine significantly different metabolites. First, PCA was conducted on serum samples. Slight differences in the serum metabolome were observed between HE and LE groups (Figures 1A,B). OPLS-DA and V-plot were then performed and compared to identify and characterize metabolites. The key parameters, R2 and Q2, were used for the evaluation of discrimination and predictive abilities of the models respectively. The results of 200-item permutation test (R^2^ = (0.0; 0.746), Q^2^ = (0.0; −0.582)) demonstrated the validity and stability of the fitted OPLS-DA model. As shown in Figures 1C,D, significant differences in serum samples were observed between HE and LE groups. Biomarkers that differed between the two groups were identified to be involved in four main pathways based on metabolic pathway analysis, including 1) biosynthesis of unsaturated fatty acids; 2) glycerolipid metabolism; 3) citrate cycle; and 4) galactose metabolism (Figure 2).

### Significantly Disturbed Metabolites Between HE Group and LE Group

**FIGURE 3:**
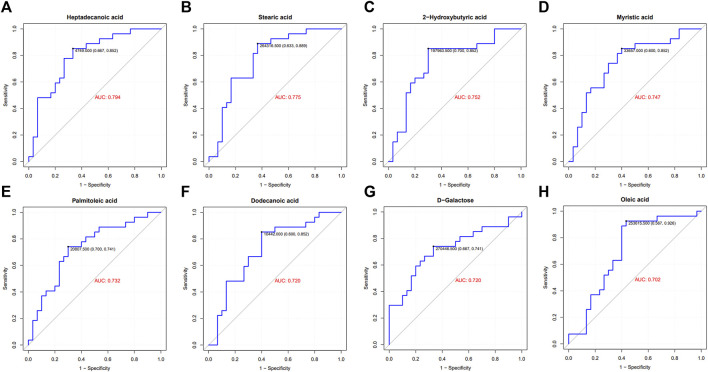
ROC curve and area under the curve (AUC) of differential metabolites. GHD patients were grouped by clinic efficacy to calculate the value of serum metabolites for the diagnosis of GHD. The ROC curve and AUC of heptadecanoic acid **(A)**, stearic acid **(B)**, 2-hydroxybutyric acid **(C)**, myristic acid **(D)**, palmitoleic acid **(E)**, dodecanoic acid **(F)**, D-galactose **(G)** and oleic acid **(H)**.

**FIGURE 4:**
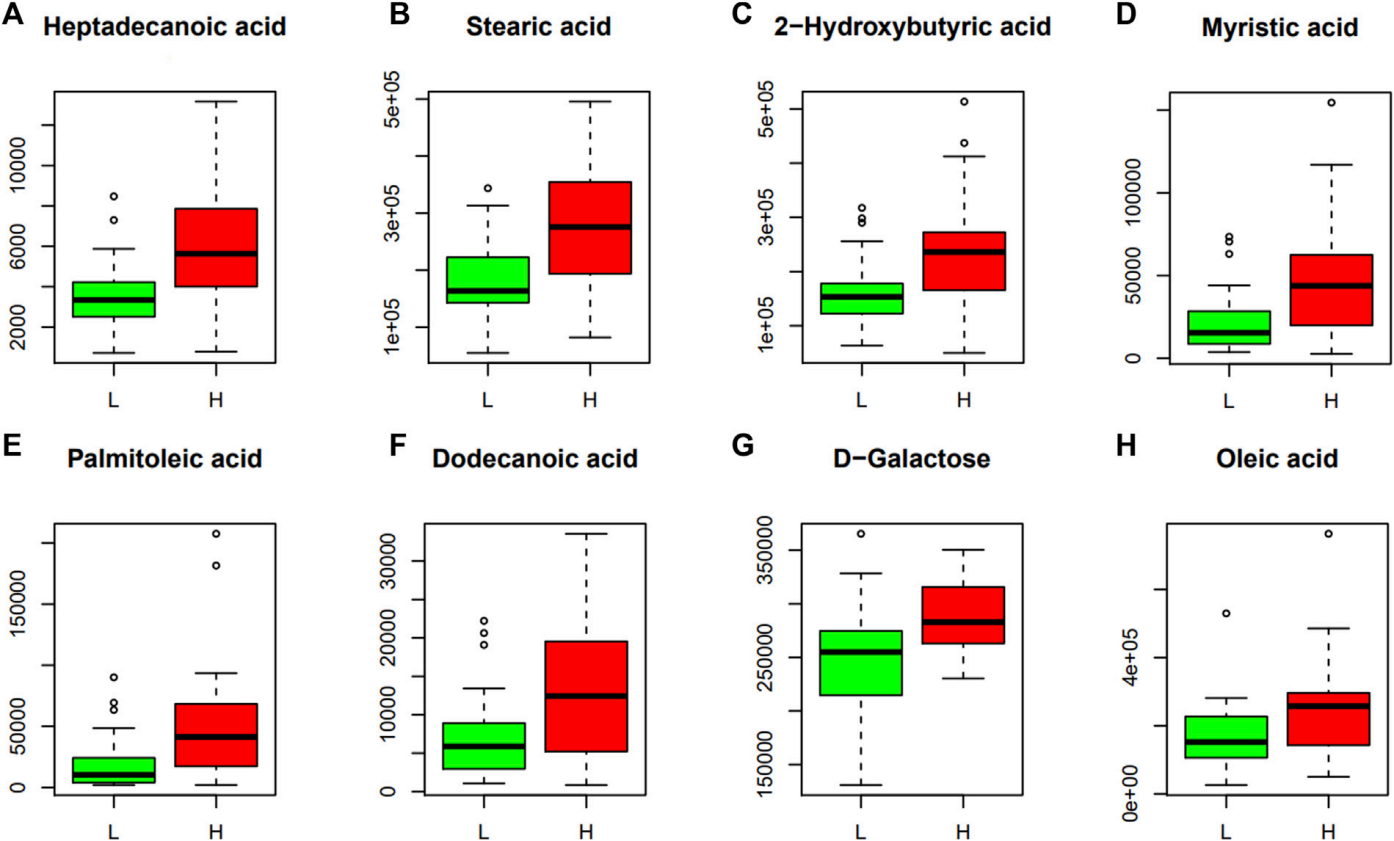
Discriminant metabolites obtained with Mann-Whitney *U* test. The resulted metabolites obtained are shown and expressed on the y axes of the graphs as the relative level of peak signals. The serum levels of heptadecanoic acid **(A)**, stearic acid **(B)**, 2-hydroxybutyric acid **(C)**, myristic acid **(D)**, palmitoleic acid **(E)**, dodecanoic acid **(F)**, D-galactose **(G)** and oleic acid **(H)**. L, LE group; H, HE group. The data are expressed as the mean ± standard deviation (n = 28 HE group; n = 29 LE group).

**FIGURE 5:**
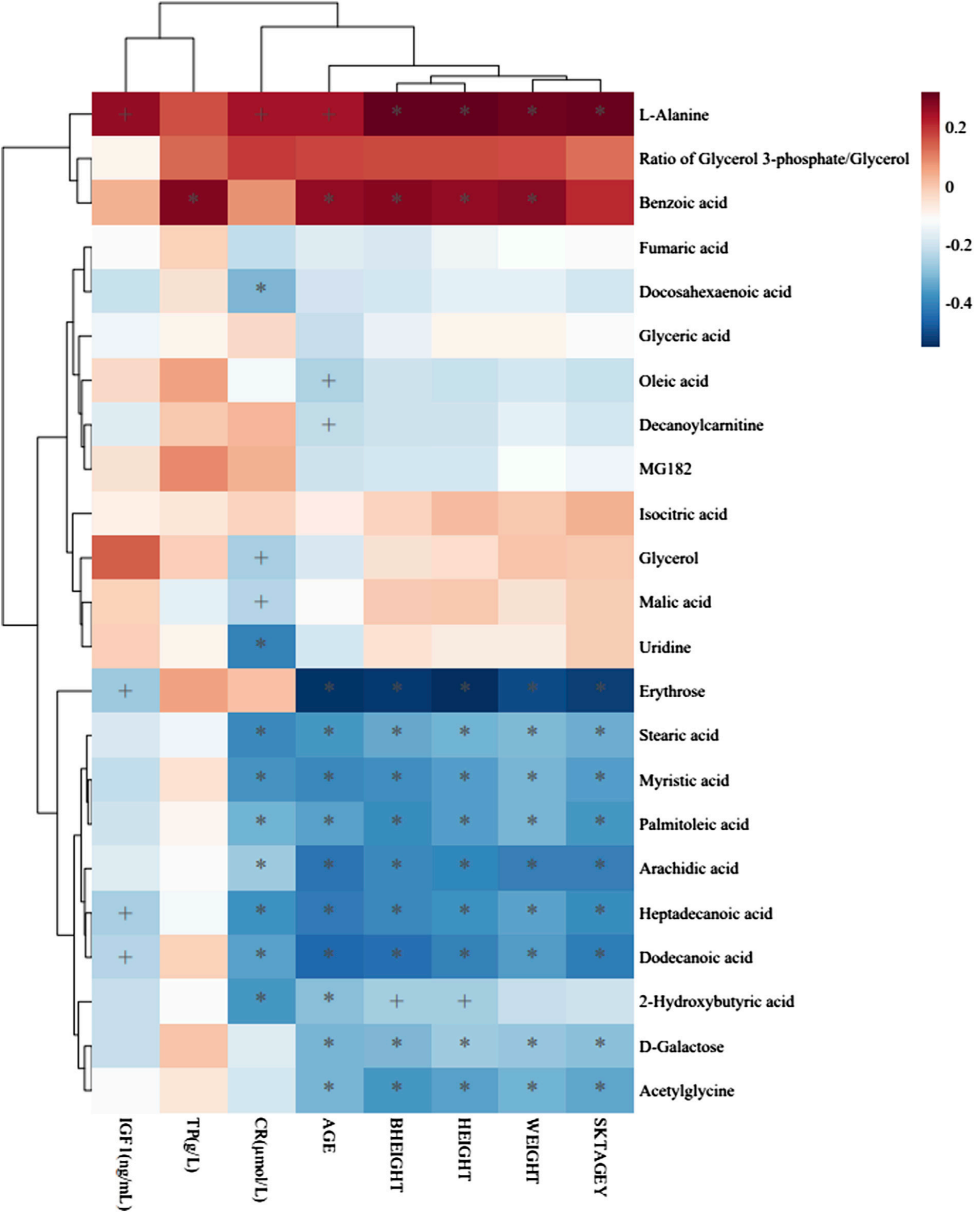
Heatmap of association between metabolites and clinical indicators. The r values are represented by gradient colors, where red and blue

To identify distinct biomarkers that may be associated with clinical efficacy among thousands of variables, a comparison was conducted between the HE and LE groups of patients. The metabolite profiles of all serum samples at baseline were analyzed to determine the relative levels of the metabolites based on GC-TOFMS analyses. A total of 119 putative metabolites were annotated, and 23 of the 119 annotated metabolites were identified (Table 2). The endogenous metabolite contributing most to the classification between the HE group and LE group was screened out using multiple criteria, including VIP value, FC value, *p*-value, and AUC value. The variables with VIP > 1 (Figure 1D), FC > 1 (Table 3), AUC > 0.700 (Figure 3), and *p* < 0.05 (Figure 4) indicated eight differential metabolites, including heptadecanoic acid, stearic acid, 2-hydroxybutyric acid, myristic acid, palmitoleic acid, D-galactose, dodecanoic acid, and oleic acid. Upregulation of metabolites was observed in the HE group compared to the LE group. Compared to the LE group, the levels of several metabolites showed an upward trend in the HE group, including heptadecanoic acid, stearic acid, 2-hydroxybutyric acid, myristic acid, palmitoleic acid, D-galactose, dodecanoic acid, and oleic acid (Table 3). We correlated the serum biochemical index changes observed in the baseline clinical characteristics of GHD patients with metabolites in HE and LE groups. A heatmap was generated to show the associations between clinical index and metabolites in the two groups (Figure 5). CR, age, bheight, height, weight, and sktagey showed strong negative correlations with heptadecanoic acid, stearic acid, myristic acid, palmitoleic acid, and dodecanoic acid. Negative correlations also were observed between the CR and age with pyroglutamic acid. A similar trend was observed between the age, bheight, height, weight, sktagey, and D-galactose.

**TABLE 2:** The identified metabolites with p < 0.05 in HE group compared to LE group at baseline^.^

Metabolites	^a^FC	^b^ *p*.value
Heptadecanoic acid	1.687	8.40E-05
Stearic acid	1.684	2.50E-04
2-Hydroxybutyric acid	1.54	8.80E-04
Myristic acid	2.836	1.10E-03
Palmitoleic acid	3.987	2.30E-03
D-Galactose	1.11	4.00E-03
Dodecanoic acid	2.115	4.00E-03
Malic acid	1.204	6.40E-03
Oleic acid	1.692	8.20E-03
Ratio of Glycerol 3-phosphate/Glycerol	0.566	1.30E-02
Uridine	1.593	1.50E-02
Acetylglycine	1.22	2.20E-02
Isocitric acid	1.815	2.50E-02
Glycerol	1.333	2.60E-02
L-Alanine	0.92	2.80E-02
Glyceric acid	1.61	2.80E-02
Decanoyl carnitine	1.298	3.10E-02
Benzoic acid	0.87	3.30E-02
Fumaric acid	1.354	3.30E-02
MG182	1.587	3.40E-02
Docosahexaenoic acid	1.299	3.90E-02
Arachidic acid	1.493	4.10E-02
Erythrose	4.918	4.40E-02

a FC, FC (fold change)-value. FC, value is the multiple difference of the metabolite concentration between samples. A value less than 1 means that the metabolite content in HE, group is lower than that in LE, group, and a value greater than 1 means that the metabolite content in HE, group is higher than that in LE, group.

b p, p-value, which is obtained from Mann-Whitney U test, p < 0.05 means the difference is statistically significant.

**TABLE 3:** Statistically significant metabolites in serum samples of HE group versus LE group comparison.

Metabolites	LE group	HE group	^a^FC	^b^VIP	^c^ *p*.value	Formula	^d^HMDB ID	HE/LE
Heptadecanoic acid	3,589.11 (1761.33)	6,194.47 (2,885.30)	1.69	2.98	8.40 × 10−5	C17H34O2	HMDB02259	↑
Stearic acid	182,390.78 (65,213.65)	280,726.2 (109,564.96)	1.68	2.87	2.50 × 10−4	C18H36O2	HMDB00827	↑
2-Hydroxybutyric acid	158,827.15 (67,109.12)	242,265.37 (106,426.26)	1.54	2.98	8.80 × 10−4	C4H8O3	HMDB00008	↑
Myristic acid	22,604.22 (19,671.17)	47,192.23 (33,841.80)	2.84	2.39	1.10 × 10−3	C14H28O2	HMDB00806	↑
Palmitoleic acid	20,111.44 (23,314.53)	50,034.73 (48,186.11)	3.99	1.92	2.30 × 10−3	C16H30O2	HMDB03229	↑
D-Galactose	246,084.44 (57,503.14)	285,655.97 (33,157.03)	1.11	2.38	4.00 × 10−3	C6H12O6	HMDB00143	↑
Dodecanoic acid	6,990.11 (5,881.99)	13,504.60 (8,949.79)	2.12	2.36	4.00 × 10−3	C12H24O2	HMDB00638	↑
Oleic acid	172,689.04 (99,261.28)	246,016.00 (142,341.37)	1.69	1.63	8.20 × 10−3	C18H34O2	HMDB00207	↑

a FC, FC (fold change)-value. FC, value is the multiple difference of the metabolite concentration between samples. A value less than 1 means that the metabolite content in HE, group is lower than that in LE, group, and a value greater than 1 means that the metabolite content in HE, group is higher than that in LE, group.

b VIP, variable importance in projection, the VIP>1 is considered to contribute to group classification.

c p, p-value, which is obtained from Mann-Whitney U test, p < 0.05 means the difference is statistically significant.

d HMDB ID, Human Metabolome Database ID.

## Discussion

The analysis of metabolomics provides a powerful tool for determining biomarkers that predict the effects of PEG-rhGH therapy through large-scale molecular analyses. The present study confirmed and extended previous studies by showing the favorable effects of PEG-rhGH therapy on GHD patients (Rogol et al., 2013; Luo et al., 2017). To explore the potential mechanism, changes of metabolites of GHD patients in HE and LE groups were monitored using a GC/TOFMS-based metabolomics method. Based on the results of metabolomics analysis, 8 differential metabolites were found to be closely related to growth gains following PEG-rhGH intervention on GHD children. These metabolites were heptadecanoic acid, stearic acid, 2-hydroxybutyric acid, myristic acid, palmitoleic acid, D-galactose, dodecanoic acid, and oleic acid. The related metabolic pathways involved fatty acid metabolism and energy metabolism.

**Fatty Acid Metabolism** A variety of fatty acids exist in the cells and tissues of humans, which play essential roles in endogenous substance metabolism, cell structure, and function (Judge and Dodd 2020). Neural cell membrane phospholipids, ceramides, and sphingolipids contain some longer-chain, saturated fatty acids, such as palmitic and stearic acids (Simons and Gerl, 2010). The saturated fatty acid content of these structures is related to their membrane location and their function. Moreover, myristic and palmitic acids can covalently modify several proteins involved in cell signaling and can influence fatty acid biosynthesis and metabolism by affecting the regulation of transcription factors, including SREBPs and LXR/RXR (Johnson et al., 1994; Mitchell et al., 2006; Calder, 2015). Previous studies have shown that fatty acids were reduced in GHD patients and became normalized upon rhGH treatment, which might raise the level of total and LDL cholesterol Cuneo et al., 1993; Russell-Jones et al., 1994; Beshyah et al., 1995; Mensink et al., 2003; Molitch et al., 2011). In the present study we showed that stearic acid, myristic acid, palmitoleic acid, and dodecanoic acid were significantly increased in the GHD patients of high clinical efficacy. These results were consistent with previous studies, indicating that fatty acid metabolism may be related to the improvement of growth and development in children with GHD. Moreover, oleic acid is a monounsaturated acid of the ω-9 series in serum, which is present endogenously in the organism and also can be obtained from the diet. Oleic acid is the most prevalent dietary fatty acid in many individuals, and it acts as a neurotrophic factor in neuronal growth (Rodríguez-Rodríguez et al., 2004; Polo-Hernández et al., 2010). There is a direct association between the exogenous administration of oleic acid and brain development in humans (Polo-Hernández et al., 2010). Interestingly, it was found that oleic acid has beneficial effects by preventing lipotoxicity, increasing mitochondrial fatty acid oxidation (Coll et al., 2008), and preventing the desensitization of human growth hormone secretagogue receptors (Delhanty et al., 2010). The present study’s results revealed a significant increase in oleic acid in the high efficacy group. More importantly, oleic acid is a biological metabolite implicated in brain growth and development, which is consistent with our GC/TOFMS-based metabolomics study. However, there is evidence that a higher intake of saturated fatty acid can increase body fat and inflammatory biomarkers for inducing incident type 2 diabetes and obesity-related diseases (Cuneo et al., 1993). Several observational studies have investigated the relationship between saturated fatty acids and inflammation in humans and have found that lauric, myristic, and palmitic acids can induce insulin resistance and can promote inflammation (Innes and Calder, 2018); however, oleic acid has the ability to reduce the inflammatory effects of long-chain, saturated fatty acids in human aortic endothelial cells (Harvey et al., 2010; Schenkel and Bakovic, 2014). The published literature is not entirely consistent with these findings, most likely because each type of fatty acid has unique effects on human metabolism. Further investigation is needed to identify the exact mechanisms involved in the function and role of fatty acid metabolism. Our results implied that regulation of fatty acid metabolism may be one of the possible mechanisms by which PEG-rhGH exerts different therapeutic effects in improving GHD. Traditionally, most interest in the health impact of fatty acids has been related to metabolic diseases and inflammatory diseases. It is now clear that they are also related to the difference in the clinical efficacy of PEG-rhGH replacement therapy.

**Energy Metabolism** Fatty acid oxidation becomes important in times of limited glucose availability. In this context good energy substrate is provided by fatty acids, which can be used to generate energy in most aerobic tissues except for the brain. Furthermore, fatty acids (palmitoleic acid and oleic acid) have distinct ways of regulating energy homeostasis (Innes and Calder, 2018). D-Galactose is completely metabolized upon first pass (from the gut) through the liver, where it is converted into glucose, lactate, glycogen, and lipids (Brouns, 2017; Gonzalez et al., 2017). Glucose is broken down to pyruvic acid, and then pyruvic acid is decarboxylated to acetyl-CoA, which is the source for the tricarboxylic acid cycle (TCA) (Akram, 2014). This cycle is an important energy-producing pathway used in eukaryotes and provides many intermediates required for gluconeogenesis and lipogenesis. In the present study the HE group showed an upward trend of the level of serum D-galactose compared to the LE group, suggesting another possible mechanism for the difference in the clinical efficacy of PEG-rhGH treatment associated with altered energy metabolism.

Our data highlighted that the clinical efficacy of PEG-rhGH treatment was associated with individual factors in GHD patients, including age, sex, and height, which suggests that individualized treatment of PEG-rhGH is needed. Bone age has been accepted as an important criterion for assessing growth and development. Patients with younger bone age before puberty can grow 8–14 cm per year using rhGH treatment (Yuen et al., 2019). As the skeletal age increases (LE group 6.81 ± 2.53 vs. HE group 4.79 ± 2.15), the growth rate of subjects will gradually decrease after PEG-rhGH treatment. Similarly, the sex-related difference is an important contributor to the efficacy of PEG-rhGH. Following general opinion, the first menarche of Asian women is estimated to be 12–16 years, and the annual growth rate is about 6–9 cm (Yang et al., 2017). At the end of the second menarche, the bone age is basically closed, possibly reflecting alterations in ovarian hormone levels *in vivo* combined with earlier developmental risks in girls. The first signs of the voice mutation can be fixed at the age of 10–11 years as the male progressed through puberty (Boskey and Coleman, 2010). Indeed, higher levels of the hormone are known to interfere with the physiological pathways of growth hormone, affecting not only growth rate but also PEG-rhGH efficacy. We note also that older participants in the LE group exhibited lower than their younger counterparts in the HE group in terms of clinical efficacy after PEG-rhGH administration. The stearic acid, myristic acid, palmitoleic acid, heptadecanoic acid, dodecanoic acid, 2-hydroxybutyric acid, and D-galactose were negatively associated with subjects’ age and had higher concentrations in the samples of the HE group. A similar trend also was observed with stearic acid, myristic acid, palmitoleic acid, heptadecanoic acid, dodecanoic acid, and D-galactose and the height and weight of GHD patients. We demonstrated that the age, height, and weight of GHD patients might have an association with a fatty acid level in serum, and we observed a possible correlation between the fatty acid level and the difference of clinical efficacy of PEG-rhGH replacement, wherein stearic acid, myristic acid, palmitoleic acid, heptadecanoic acid, dodecanoic acid, 2-hydroxybutyric acid, and D-galactose were suggested as biomarkers for predicting the PEG-rhGH efficacy.

This is a well-designed clinical study with strict inclusion and exclusion criteria although the number of qualified participants was small in this study. Thus, further large-scale and multi-center-based studies are needed for further validation. Moreover, non-targeted metabolomics studies contributed to biomarker discovery initially, so further targeted and quantitative analysis of differential metabolites are required in the future.

## Conclusion

In conclusion, our results revealed for the first time the identity of important metabolites and pathways that contribute to predicting the efficacy of PEG-rhGH therapy. Several predictive marker candidates for the effects of PEG-rhGH treatment were identified, including heptadecanoic acid, stearic acid, 2-hydroxybutyric acid, myristic acid, palmitoleic acid, D-galactose, dodecanoic acid, and oleic acid. The results revealed a strong association between the serum metabolic profiles and the clinical efficacy of PEG-rhGH therapy, and this association was most pronounced in fatty acids. The mechanisms are likely to be involved in fatty acid metabolism and energy metabolism. The current study suggested fatty acid as the potential predictive marker for PEG-rhGH treatment effects. These discoveries enabled a better understanding of the mechanism of PEG-rhGH and may lead to the development of metabolic biomarkers and novel therapeutic strategies for individualized treatment of PEG-rhGH.

## Data Availability

The original contributions presented in the study are included in the article/Supplementary Material, further inquiries can be directed to the corresponding authors.
