# Supplementary material for: Metabolomic Differential Compounds Reflecting the Clinical Efficacy of Polyethylene Glycol Recombinant Human Growth Hormone in the Treatment of Childhood Growth Hormone Deficiency
Source: Front Pharmacol. 2022 Apr 27;13:864058. doi: 10.3389/fphar.2022.864058 (PMC9092529; doi:10.3389/fphar.2022.864058)
Supplement: Supplementary file 1 [file Table1.DOCX]

**TABLE 1** Baseline clinical characteristics

| **Item** | **LE group** | **HE group** | ^a^**FC** | ^*^***P*** |
| --- | --- | --- | --- | --- |
| ^¶^BHEIGHT (cm) | 119.27 (13.79) | 108.19 (10.12) | 0.877 | 1.10×10^-3^ |
| HEIGHT (cm) | 121.8 (13.19) | 110.98 (10.56) | 0.885 | 1.70×10^-3^ |
| WEIGHT (kg) | 25.8 (7.38) | 20.18 (5.96) | 0.738 | 2.90×10^-3^ |
| BONE AGE | 6.81 (2.53) | 4.79 (2.15) | 0.571 | 3.30×10^-3^ |
| AGE (year) | 11.93 (2.79) | 10 (2.29) | 0.833 | 6.70×10^-3^ |
| BMI | 16.99 (2.38) | 16.06 (2.24) | 0.920 | 0.067 |
| ^#^SEX_STD | 1.19 (0.40) | 1.3 (0.47) | 1.000 | 0.486 |
| **Liver Function** | | | | |
| PHOS (mmol/L) | 1.57 (0.19) | 1.56 (0.16) | 1.039 | 0.947 |
| ALP (IU/L) | 219.93 (71.59) | 226.76 (58.69) | 1.138 | 0.344 |
| TBIL (μmol/L) | 8.41 (5.34) | 7.92 (2.95) | 1.054 | 0.966 |
| ALT (IU/L) | 15.14 (6.02) | 17.02 (7.76) | 1.100 | 0.321 |
| AST (IU/L) | 29 (8.42) | 31.17 (7.30) | 1.089 | 0.174 |
| ALB (g/L) | 44.06 (2.96) | 43.56 (2.63) | 1.023 | 0.566 |
| TP (g/L) | 70.14 (3.95) | 66.95 (4.82) | 0.973 | 1.50×10^-2^ |
| **Renal Function** | | | | |
| SG | 1.02 (0.01) | 1.02 (0.00) | 1.001 | 0.611 |
| BUN (mmol/L) | 4.58 (0.97) | 4.86 (1.25) | 0.980 | 0.457 |
| PH | 6.4 (0.86) | 6.25 (0.74) | 0.923 | 0.442 |
| CR (μmol/L) | 47.93 (7.81) | 41.24 (12.94) | 0.856 | 1.80×10^-2^ |
| UWBC | 1.05 (1.09) | 1.38 (1.85) | 1.000 | 0.890 |
| URBC | 2.1 (2.31) | 2.24 (3.46) | 0.763 | 0.489 |
| **Blood Lipid** | | | | |
| TG (mmol/L) | 0.91 (0.62) | 0.84 (0.34) | 1.013 | 0.954 |
| TC (mmol/L) | 4.09 (0.68) | 4.29 (0.99) | 1.040 | 0.915 |
| HDL (mmol/L) | 1.52 (0.28) | 1.45 (0.30) | 0.980 | 0.561 |
| LDL (mmol/L) | 2.25 (0.54) | 2.48 (0.77) | 1.039 | 0.448 |
| **Blood Glucose and Hormone** | | | | |
| HBA1C (%) | 5.26 (0.34) | 5.31 (0.40) | 1.000 | 0.574 |
| GLU (mmol/L) | 5.04 (1.06) | 4.81 (0.40) | 0.966 | 0.476 |
| CORT (nmol/L) | 97.68 (139.49) | 130.75 (223.92) | 1.196 | 0.634 |
| ACTH (pmol/L) | 31.63 (26.69) | 25.25 (18.18) | 1.043 | 0.595 |
| INS (mIU/L) | 14 (22.22) | 9.27 (8.01) | 0.805 | 0.574 |
| **Thyroid** | | | | |
| TSH (mIU/L) | 2.91 (1.20) | 2.81 (1.63) | 0.936 | 0.560 |
| T3 (nmol/L) | 1.82 (0.49) | 2.03 (0.50) | 1.319 | 0.057 |
| T4 (nmol/L) | 70.07 (51.35) | 82.65 (51.77) | 1.135 | 0.242 |
| **Blood Routine** | | | | |
| RBC (×10^12^/L) | 4.7 (0.40) | 4.62 (0.41) | 0.951 | 0.212 |
| WBC (×10^9^/L) | 7.27 (2.16) | 6.89 (2.28) | 0.949 | 0.421 |
| HCT | 27.06 (20.39) | 32.8 (13.12) | 1.012 | 0.844 |
| HB (g/L) | 128.54 (11.64) | 124.44 (9.16) | 0.962 | 0.076 |
| PLT (×10^9^/L) | 283.08 (69.01) | 293.3 (63.42) | 1.007 | 0.588 |
| **Others** | | | | |
| IGF-1 (ng/mL) | 174.63 (87.20) | 111.4 (69.84) | 0.595 | 4.80×10^-3^ |
| IGFBP-3 (ng/mL) | 3.54 (0.95) | 2.96 (1.09) | 0.880 | 0.076 |
| CAL (mmol/L) | 2.35 (0.19) | 2.38 (0.13) | 1.004 | 0.848 |

*^a^FC, FC (fold change)-value. FC value is the multiple difference of the metabolite concentration between samples. A value less than 1 means that the metabolite content in HE group is lower than that in LE group, and a value greater than 1 means that the metabolite content in HE group is higher than that in LE group; ^*^P, p-value, which is obtained from Mann-Whitney U test, p < 0.05 means the difference is statistically significant; ^#^SEX_STD, 1 represents male and 2 represents female for gender comparison; ^¶^BHEIGHT, height one year prior to the treatment.*

**TABLE 2** The annotated metabolites with *p* < 0.05 in HE group compared to LE group at baseline.

| **Metabolites** | ^a^**FC** | ^*^***p.*value** |
| --- | --- | --- |
| Heptadecanoic acid | 1.687 | 8.40E-05 |
| Stearic acid | 1.684 | 2.50E-04 |
| 2-Hydroxybutyric acid | 1.54 | 8.80E-04 |
| Myristic acid | 2.836 | 1.10E-03 |
| Palmitoleic acid | 3.987 | 2.30E-03 |
| D-Galactose | 1.11 | 4.00E-03 |
| Dodecanoic acid | 2.115 | 4.00E-03 |
| Malic acid | 1.204 | 6.40E-03 |
| Oleic acid | 1.692 | 8.20E-03 |
| Ratio of Glycerol 3-phosphate/Glycerol | 0.566 | 1.30E-02 |
| Uridine | 1.593 | 1.50E-02 |
| Acetylglycine | 1.22 | 2.20E-02 |
| Isocitric acid | 1.815 | 2.50E-02 |
| Glycerol | 1.333 | 2.60E-02 |
| L-Alanine | 0.92 | 2.80E-02 |
| Glyceric acid | 1.61 | 2.80E-02 |
| Decanoyl carnitine | 1.298 | 3.10E-02 |
| Benzoic acid | 0.87 | 3.30E-02 |
| Fumaric acid | 1.354 | 3.30E-02 |
| MG182 | 1.587 | 3.40E-02 |
| Docosahexaenoic acid | 1.299 | 3.90E-02 |
| Arachidic acid | 1.493 | 4.10E-02 |
| Erythrose | 4.918 | 4.40E-02 |

*^a^FC, FC (fold change)-value. FC value is the multiple difference of the metabolite concentration between samples. A value less than 1 means that the metabolite content in HE group is lower than that in LE group, and a value greater than 1 means that the metabolite content in HE group is higher than that in LE group; ^*^p, p-value, which is obtained from Mann-Whitney U test, p < 0.05 means the difference is statistically significant.*

**TABLE 3** Statistically significant metabolites in serum samples of HE group versus LE group comparison.

| **Metabolites** | **LE group** | **HE group** | ^a^**FC** | ^b^**VIP** | ^*^***p.*value** | **Formula** | ^c^**HMDB ID** | **HE/**  **LE** |
| --- | --- | --- | --- | --- | --- | --- | --- | --- |
| Heptadecanoic acid | 3589.11 (1761.33) | 6194.47 (2885.30) | 1.69 | 2.98 | 8.40×10^-5^ | C_17_H_34_O_2_ | HMDB02259 | **↑** |
| Stearic acid | 182390.78 (65213.65) | 280726.2 (109564.96) | 1.68 | 2.87 | 2.50×10^-4^ | C_18_H_36_O_2_ | HMDB00827 | **↑** |
| 2-Hydroxybutyric acid | 158827.15 (67109.12) | 242265.37 (106426.26) | 1.54 | 2.98 | 8.80×10^-4^ | C_4_H_8_O_3_ | HMDB00008 | **↑** |
| Myristic acid | 22604.22 (19671.17) | 47192.23 (33841.80) | 2.84 | 2.39 | 1.10×10^-3^ | C_14_H_28_O_2_ | HMDB00806 | **↑** |
| Palmitoleic acid | 20111.44 (23314.53) | 50034.73 (48186.11) | 3.99 | 1.92 | 2.30×10^-3^ | C_16_H_30_O_2_ | HMDB03229 | **↑** |
| D-Galactose | 246084.44 (57503.14) | 285655.97 (33157.03) | 1.11 | 2.38 | 4.00×10^-3^ | C_6_H_12_O_6_ | HMDB00143 | **↑** |
| Dodecanoic acid | 6990.11 (5881.99) | 13504.60 (8949.79) | 2.12 | 2.36 | 4.00×10^-3^ | C_12_H_24_O_2_ | HMDB00638 | **↑** |
| Oleic acid | 172689.04 (99261.28) | 246016.00 (142341.37) | 1.69 | 1.63 | 8.20×10^-3^ | C_18_H_34_O_2_ | HMDB00207 | **↑** |

*^a^FC, FC (fold change)-value. FC value is the multiple difference of the metabolite concentration between samples. A value less than 1 means that the metabolite content in HE group is lower than that in LE group, and a value greater than 1 means that the metabolite content in HE group is higher than that in LE group; ^b^VIP, Variable importance in projection, the VIP>1 is considered to contribute to group classification; ^*^p, p-value, which is obtained from Mann-Whitney U test, p < 0.05 means the difference is statistically significant; ^c^HMDB ID, Human Metabolome Database ID.*
